# Advances in the structural basis for angiotensin-1 converting enzyme (ACE) inhibitors

**DOI:** 10.1042/BSR20240130

**Published:** 2024-08-05

**Authors:** K. Ravi Acharya, Kyle S. Gregory, Edward D. Sturrock

**Affiliations:** 1 Department of Life Sciences, University of Bath, Claverton Down, Bath BA2 7AY, U.K.; 2 Department of Integrative Biomedical Sciences, Institute of Infectious Disease and Molecular Medicine, University of Cape Town, Observatory 7925, Cape Town, Republic of South Africa

**Keywords:** enzymology, inhibitor design, structural biology

## Abstract

Human somatic angiotensin-converting enzyme (ACE) is a key zinc metallopeptidase that plays a pivotal role in the renin–angiotensin–aldosterone system (RAAS) by regulating blood pressure and electrolyte balance. Inhibition of ACE is a cornerstone in the management of hypertension, cardiovascular diseases, and renal disorders. Recent advances in structural biology techniques have provided invaluable insights into the molecular mechanisms underlying ACE inhibition, facilitating the design and development of more effective therapeutic agents. This review focuses on the latest advancements in elucidating the structural basis for ACE inhibition. High-resolution crystallographic studies of minimally glycosylated individual domains of ACE have revealed intricate molecular details of the ACE catalytic N- and C-domains, and their detailed interactions with clinically relevant and newly designed domain-specific inhibitors. In addition, the recently elucidated structure of the glycosylated form of full-length ACE by cryo-electron microscopy (cryo-EM) has shed light on the mechanism of ACE dimerization and revealed continuous conformational changes which occur prior to ligand binding. In addition to these experimental techniques, computational approaches have also played a pivotal role in elucidating the structural basis for ACE inhibition. Molecular dynamics simulations and computational docking studies have provided atomic details of inhibitor binding kinetics and energetics, facilitating the rational design of novel ACE inhibitors with improved potency and selectivity. Furthermore, computational analysis of the motions observed by cryo-EM allowed the identification of allosteric binding sites on ACE. This affords new opportunities for the development of next-generation allosteric inhibitors with enhanced pharmacological properties. Overall, the insights highlighted in this review could enable the rational design of novel ACE inhibitors with improved efficacy and safety profiles, ultimately leading to better therapeutic outcomes for patients with hypertension and cardiovascular diseases.

## Introduction

### Angiotensin-1 converting enzyme (ACE)

The renin–angiotensin–aldosterone system (RAAS) is a complex and tightly regulated hormonal system that influences blood pressure, fluid and electrolyte balance, and vascular tone. The RAAS consists of a series of enzymatic and hormonal interactions that ultimately lead to the production of angiotensin II (Ang II), a potent vasoconstrictor. The cascade begins with the release of renin from juxtaglomerular cells in the kidneys in response to various stimuli, such as low blood pressure or decreased sodium levels. Renin acts on angiotensinogen, a precursor protein produced by the liver, converting it into angiotensin I (Ang I, DRVYIHPFHL). This inactive decapeptide is further converted into the active octapeptide angiotensin II (Ang II, DRVYIHPF) by the crucial enzyme angiotensin-1 converting enzyme (ACE, EC 3.4.15.1). This conversion has profound implications for blood pressure regulation and various cardiovascular and renal functions. Ang II exerts potent vasoconstrictive effects by acting on the angiotensin II receptor Type I (AT_1_R) of smooth muscle cells, particularly in the arterioles. This vasoconstriction results in an increase in peripheral vascular resistance, contributing to elevated blood pressure.

The primary physiological role of ACE in blood pressure regulation is underscored by its involvement in the conversion of Ang I to Ang II, encompassing the modulation of various vasoactive peptides, including kinins like bradykinin (BK) (PPGFSPFR), substance P, and acetylated Ser-Asp-Lys-Pro (Ac-SDKP). Because of its promiscuity as an enzyme, ACE and its peptide substrates and products affect many physiological processes, including blood pressure control, haematopoiesis, reproduction, renal development, renal function, and the immune response [1,2] (for reviews, see Acharya et al. [3]; Bernstein et al. [4]; Arendse et al. [5]; Rao et al. [6]). This versatility makes ACE a pivotal enzyme with implications for health and disease.

Apart from the action of Ang II on the AT_1_R, Ang II further stimulates the release of aldosterone from the adrenal cortex. Aldosterone promotes sodium retention in the kidneys, leading to increased water reabsorption and expansion of extracellular fluid volume. The combined effects of vasoconstriction and fluid retention contribute to the overall elevation of blood pressure, emphasizing the central role of ACE in the pathophysiology of hypertension [7]. As well as its hypertensive effects on blood vessels, Ang II also acts as a growth factor, promoting thickening of blood vessel walls, thereby exacerbating atherosclerosis. Inhibition of ACE and the RAAS by ACE inhibitors reduces blood pressure, enhances cardiac function, and decelerates the progression of atherosclerosis and kidney disease. Consequently, ACE exerts profound effects on blood pressure regulation and cardiovascular function, playing a crucial role in maintaining hemodynamic stability. For these reasons, drugs that target the RAAS, such as ACE inhibitors (introduced in 1981) and Ang II receptor blockers (ARBs), are among the most important therapeutic agents available today for the treatment of hypertension, heart failure, coronary artery disease, renal insufficiency, and general atherosclerosis [8]. The development of an orally available, non-peptide ACE inhibitor was first achieved by Cushman et al. [9] and Ondetti et al. [10].

### Mechanism of action of ACE inhibitors

ACE inhibitors exert their therapeutic effects by competitively inhibiting the enzymatic activity of ACE, with *K*_i_ values ranging between 10^−10^ and 10^−11^ M. ACE catalyses the cleavage of pairs of amino acids from the carboxy-terminal end of numerous peptide substrates. Among these substrates, the conversion of Ang I to Ang II and the degradation of BK are considered pivotal functions of ACE.

**Figure 1:**
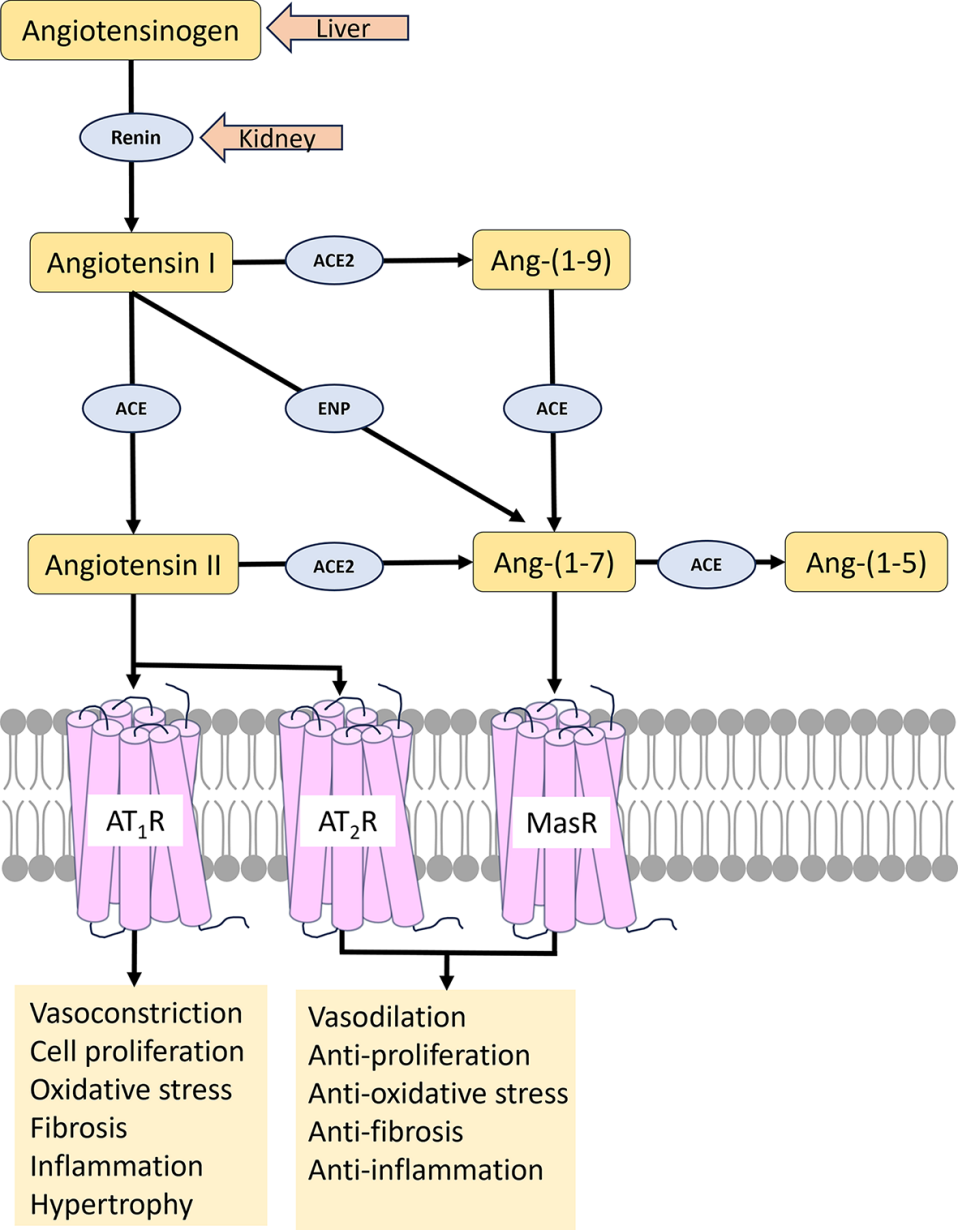
An overview of the renin–angiotensin–aldosterone system (RAAS) A complex pathway of peptides converted to active hormones (orange rectangles) by peptidases (blue ovals) and key receptors (pink cylinders) provide a number of targets for therapeutic intervention. ACE, angiotensin-1 converting enzyme; ACE2, angiotensin-converting enzyme 2; AT_1_R, angiotensin type 1 receptor: AT_2_R, angiotensin type 2 receptor; MasR, Mas receptor for Ang-(1-7); ENP, endopeptidases. The effect of AT_1_R, AT_2_R, and MasR stimulation are shown under each receptor.

BK is a vasodilator peptide hormone with anti-inflammatory properties that is produced as part of the kallikrein–kinin cascade. ACE inhibition results in decreased Ang II levels (reduced vasoconstriction) and increased BK levels (enhanced vasodilation), collectively leading to a greater reduction in blood pressure compared with the effect of inhibiting the action of Ang II alone. This dual action on the RAAS and the kallikrein–kinin pathway contributes to the unique therapeutic profile of ACE inhibitors, which is distinct from ARBs that solely target the AT_1_R (Figure 1).

Currently available ACE inhibitors are non-peptide analogues of Ang I, which function by reducing ACE enzymatic activity, thereby diminishing Ang II production. Consequently, blood vessels dilate, blood pressure decreases, and cardiac workload is alleviated. Furthermore, ACE inhibition slows the progression of kidney disease associated with hypertension or diabetes.

### Molecular and structural features of ACE domains: X-ray crystallographic studies

**Figure 2:**
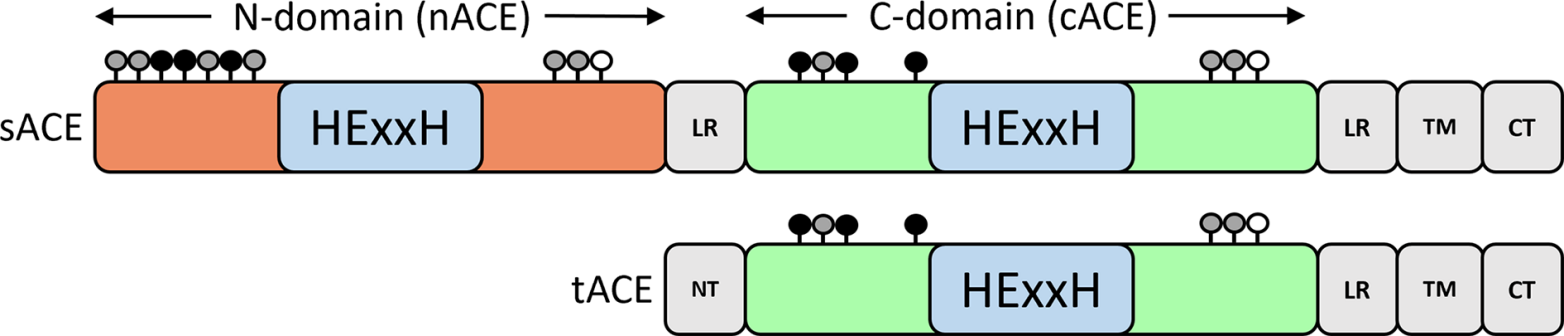
Schematic representation of the domain structure of sACE and tACE LR linker region, NT N-terminus, SR stalk region, TM transmembrane region, CT C-terminus, HExxH zinc binding histidine and catalytic glutamate conserved motif. Glycosylation is shown by the black (always glycosylated), grey (partially glycosylated) and white (not glycosylated) circles.

Significant contributions to understanding the structure-function relationship of ACE have been made using X-ray crystallography and cryo-EM. There are two isoforms of human ACE. In somatic tissues, ACE exists as a monomeric, type I transmembrane glycoprotein comprising 1306 amino acids in its mature form. A sequence of 22 hydrophobic amino acids, located near the carboxy terminus, functions as the transmembrane domain anchoring ACE to the cell surface. This configuration gives rise to a 28-residue cytosolic domain and a 1277-residue glycosylated (30% by weight) extracellular domain (Figure 2). sACE localizes primarily on the plasma membrane of endothelial, absorptive epithelial, and neuroepithelial cells. Notably, ACE belongs to a family of proteins that undergo cleavage, a process controlled by various mechanisms, including protein kinase C activation [11].

Following cleavage, a soluble form of sACE is released that comprises a mature single large polypeptide chain of 1277 amino acids, corresponding to the extracellular domain of the full-length membrane protein. The concentration of sACE in human plasma typically ranges from 36 to 288 ng/ml (260–2076 pM), approximately 200-fold higher than that of Ang I. However, soluble sACE’s impact on tissue Ang II levels is limited, with local conversion of Ang I to Ang II by endothelial ACE playing a more crucial role, particularly in proximity to AT_1_R. sACE contains two homologous catalytically active centres on each N- and C-domain, referred to as nACE and cACE, respectively (Figure 2). These domains share significant homology, with approximately 60% amino acid sequence identity. Both domains feature a HExxH zinc-binding motif that is crucial for catalytic activity.

Interestingly sACE is related to ACE2, which also regulates the RAAS and serves as a functional receptor for coronaviruses. However, ACE2 differs from sACE in ligand-binding specificity and possesses a single peptidase domain [12,13].

In germinal cells, ACE is synthesized as a lower molecular mass form, known as testis ACE (tACE), which plays a role in sperm maturation and the binding of sperm to the oviduct epithelium [14]. tACE is identical to cACE, except for a unique 36-residue sequence constituting its amino terminus (Figure 2).

**Figure 3:**
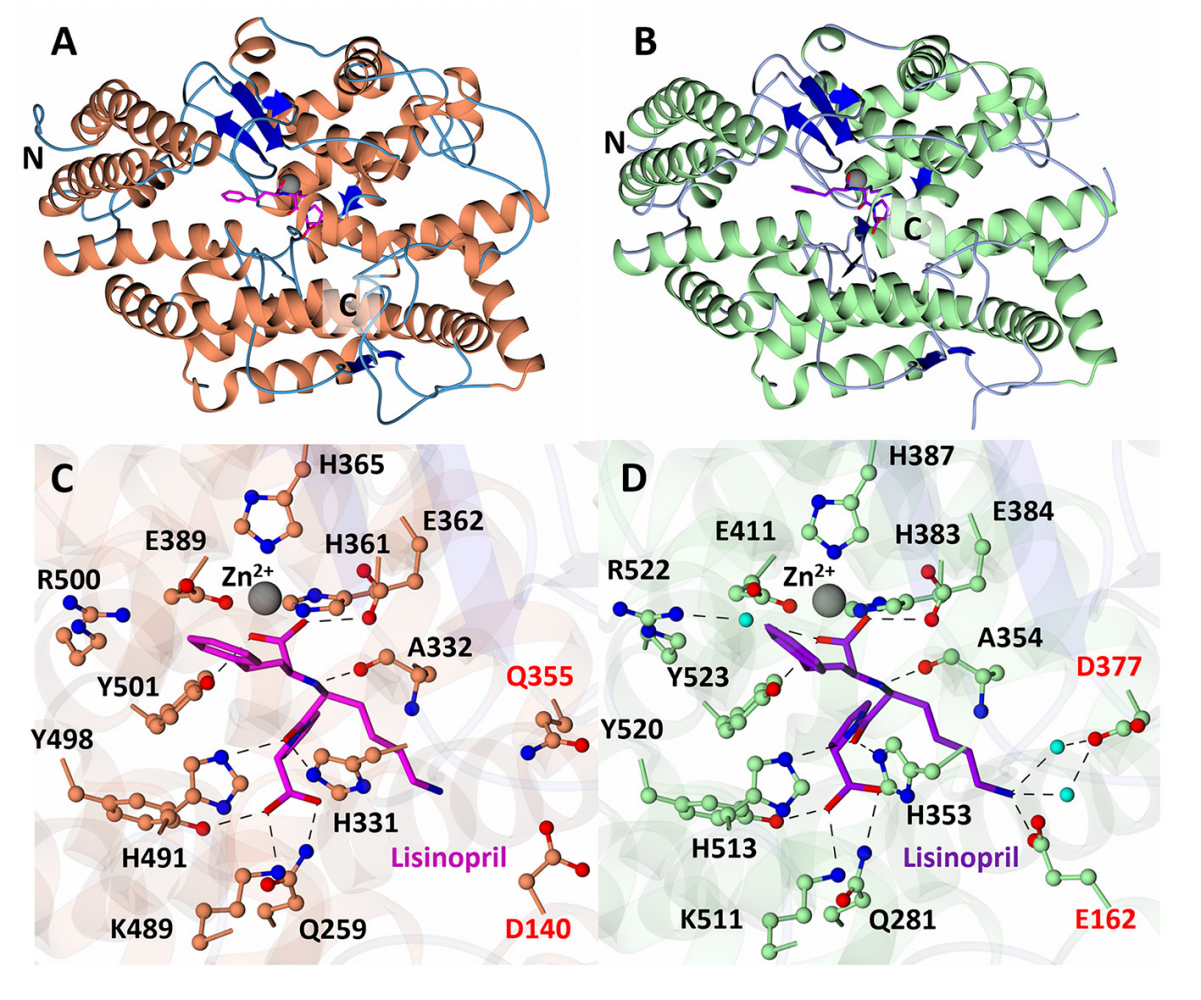
Closed structures of nACE (PDB code: 2C6N, Corradi et al., 2006) and cACE (PDB code: 1O86, Natesh et al., 2003) in complex with lisinopril Schematic representation of the overall structures of (**A**) nACE and (**B**) cACE inhibitor complexes (loop regions are shortened for clarity), with close-up view of bound lisinopril in the active site of (**C**) nACE and (**D**) cACE. Zinc ions and water molecules are depicted as grey and cyan spheres, respectively, with nACE and cACE helices coloured in orange and green, respectively. β-strands are coloured blue. Lisinopril and loop regions are coloured silver and pink for nACE and silver and purple for cACE.

The elucidation of ACE’s function has been greatly facilitated by detailed molecular structures based on high-resolution crystal structures of cACE and nACE with clinically relevant ACE inhibitors, such as lisinopril [15,16] (Figure 3). These structures have revealed the presence of a catalytic site with a zinc ion buried deep within the molecule’s central cavity, featuring a conserved HExxH zinc binding motif comprised of two histidine residues coordinating the zinc ion, along with a conserved glutamate residue. Furthermore, the identification of functionally important chloride ion binding sites in both cACE and nACE has provided insights into their enzymatic activities [15,16]. An important feature of ACE is its activation by chloride ions, which serve as an allosteric activator [17]. Chloride ions induce conformational changes that influence substrate binding, with the activity of cACE being particularly dependent on chloride ion concentration. In contrast, nACE maintains catalytic activity even at low chloride ion concentrations, suggesting physiological implications for domain-specific activity modulation by chloride ions.

Both nACE and cACE exhibit distinct but overlapping substrate specificities and physiological functions, differing in chloride dependence and glycosylation patterns [18,19]. While both domains efficiently catalyze the degradation of BK [20], knockout mouse models suggest that one domain’s cleavage of BK can compensate for the absence of the other [21]. However, cACE predominantly facilitates Ang II formation and plays a crucial role in blood pressure regulation *in vivo* [21,22]. On the other hand, nACE serves as the primary site for the clearance of the tetrapeptide Ac-SDKP, known for its anti-inflammatory and antifibrotic properties [23]. Moreover, nACE exhibits greater thermal stability and resistance to proteolysis under denaturing conditions than cACE. These differences contribute to the complexity of studying the structure of ACE.

Efforts to uncover the X-ray structure of ACE inhibitor complexes have been intensive due to their clinical importance. However, achieving crystallization of the full-length sACE protein has proved challenging. The main obstacles stem from the protein’s extensive surface glycosylation, and the flexibility of the linking chain between its two domains. Progress has been made by tackling the crystallography of cACE and nACE separately [15,16]. These studies have provided insights into the architecture and functionality of ACE. The crystal structure of cACE revealed an ellipsoidal protein shape with a central groove housing the active site, coordinated by catalytic zinc ions. The entrance to this active site is shielded by an N-terminal lid composed of three helices, which prevents large peptides from accessing the active site, and explaining ACE’s preference for small peptide substrates. Similarly, the crystal structure of nACE mirrors the architecture of cACE, featuring an ellipsoidal shape with a central groove and an N-terminal lid. However, there are differences in the lid region’s charge distribution that potentially influence substrate specificity between the domains. Additionally, differences in flexible loop arrangements further distinguish nACE from cACE.

Since these studies in the 2000s, additional X-ray crystallographic structures depicting cACE and nACE with various inhibitors have been published by the Acharya and Sturrock groups (the authors of the present review article, Table 1).

**Table 1:** Molecular and structural features of ACE domains: X-ray crystallography studies

Study	Crystal structure/s	PDB ID	Year
Structure of native cACE and cACE in complex with lisinopril	cACE (native)	1O8A	2003
	cACE in complex with lisinopril	1O86	
Structure of cACE in complex with enalaprilat and captopril	cACE in in complex with enalaprilat	1UZE	2004
	cACE in complex with captopril	1UZF	
Structure of native nACE and nACE in complex with lisinopril	nACE (native)	2C6F	2006
	nACE in complex with lisinopril	2C6N	
cACE glycosylation mutants and evidence for conserved domain movement	cACE (tACE^*^ g1,3 mutant)	2IUL	2006
	cACE (tACE^*^ g1234 mutant)	2IUX	
cACE in complex with the cACE specific inhibitor, RXPA380	cACE in complex with RXPA380	2OC2	2007
inhibition using novel ketone inhibitors of cACE	cACE in complex with ketone ACE inhibitor kAF	3BKK	2008
	cACE in complex with ketone ACE inhibitor kAW	3BKL	
Domain-selective inhibitor binding in cACE using a novel derivative of lisinopril	cACE in complex with lisinopril-W	3L3N	2010
The role of N-glycosylation in nACE and nACE in complex with the nACE specific inhibitor, RXP407	nACE in complex with RXP407	3NXQ	2010
Novel mechanism of inhibition of cACE and nACE by a highly specific phosphonic tripeptide	cACE in complex with a phosphinic tripeptide FII	2XY9	2011
	nACE in complex with phosphinic tripeptide FII	2XYD	
cACE in complex with selenium analogue of captopril	cACE in complex with a selenium analogue of captopril	2YDM	2011
Molecular recognition and regulation of cACE activity by natural inhibitory peptides	cACE in complex with Ang II peptide	4APH	2012
	cACE in complex with BPPb peptide	4APJ	
Fragment based design for the development of nACE selective ACE inhibitors	nACE in complex with 33RE	4BXK	2013
Molecular and thermodynamic mechanisms of the chloride dependent ACE	cACE mutant E403R	4C2N	2013
	cACE mutant D465T	4C2O	
	cACE mutant R522K	4C2Q	
	cACE (R522K) in complex with captopril	4C2P	
	cACE mutant R522Q	4C2R	
cACE and nACE in complex with the highly specific phosphinic tripeptide, FI	cACE in complex with phosphinic tripeptide FI	4CA5	2013
	nACE in complex with phosphinic tripeptide FI	4CA6	
Interkingdom pharmacology of ACE inhibitor phosphonates produced by actinomycetes	cACE in complex with K-26	4BZR	2014
	nACE in complex with K-26	4BZS	
Structural basis of Ac-SDKP hydrolysis by nACE	nACE in complex with Ac-SD	4UFA	2015
	nACE in complex with Lys-pro	4UFB	
The kinetic and structural characterisation of amyloid-β metabolism by ACE	nACE in complex with amyloid beta fragment 4-10	5AM8	2015
	nACE in complex with amyloid beta fragment 10-16	5AM9	
	nACE in complex with amyloid beta fragment 1-16	5AMA	
	nACE in complex with amyloid beta fragment 35-42	5AMB	
	nACE in complex with amyloid beta flurogenic fragment 4-10	5AMC	
The design and development of a potent and selective novel diprolyl derivative that binds to nACE	nACE in complex with a diprolyl inhibitor SG6 (crystal structure A)	6EN5	2018
	nACE in complex with a diprolyl inhibitor SG6 (crystal structure B)	6EN6	
cACE and nACE in complex with sampatrilat and sampatrilat-Asp - a molecular basis of domain selectivity	cACE in complex with sampatrilat	6F9T	2017
	cACE in complex with sampatrilat-Asp	6F9U	
	nACE in complex with sampatrilat	6F9V	
	nACE in complex with sampatrilat-Asp	6F9R	
cACE and nACE in complex with omapatrilat reveals multiple binding sites in cACE	cACE in complex with omapatrilat	6H5W	2018
	nACE in complex with omapatrilat	6H5X	
cACE selective ACE inhibition by bradykinin-potentiating peptide b	nACE in complex with BPPb	6QS1	2019
ACE-domain selectivity extends beyond direct interacting residues at the active site	nACE res_S2 mutant in complex with 33RE	6TT1	2019
	nACE res_S2 mutant in complex with SG6	6TT3	
	nACE res_S2 mutant in complex with omapatrilat	6TT4	
ACE open for business: structural insights into the subdomain dynamics	cACE with inserted symmetry molecule C-terminus	6ZPU	2020
	nACE ‘open’ structure	6ZPQ	
	nACE S2_S′ mutant	6ZPT	
Probing the requirements for dual cACE/Neprilysin inhibition	nACE in complex with dual ACE/NEP inhibitor AD011	7Q24	2021
	nACE in complex with dual ACE/NEP inhibitor AD012	7Q25	
	nACE in complex with dual ACE/NEP inhibitor AD013	7Q26	
	cACE in complex with dual ACE/NEP inhibitor AD011	7Q27	
	cACE in complex with dual ACE/NEP inhibitor AD012	7Q28	
	cACE in complex with dual ACE/NEP inhibitor AD013	7Q29	
Structural basis for the inhibition of ACE by fosinoprilat	cACE in complex with fosinoprilat	7Z70	2022
	nACE in complex with fosinoprilat	7Z6Z	
Structural insights into the inhibitory mechanism of ACE by IPP and VPP	nACE in complex with IPP	8QFX	2023
	nACE in complex with VPP	8QHL	

* tACE is equivalent to cACE, g stands for glycosylation, the numbers indicate the constructs are minimally glycosylated, with glycans at the corresponding positions i.e. tACE g1,3 has glycosylation at the first and third glycosylation site only.

### Design of first-generation classical ACE inhibitors

In a seminal report published in 1977, Cushman and colleagues embarked on a groundbreaking endeavour to design potent ACE inhibitors [9,10]. Their innovative approach drew upon the mechanistic parallels between ACE and carboxypeptidase A, laying the foundation for a new era in cardiovascular pharmacotherapy. This pioneering work gave birth to a cohort of drugs, including captopril, lisinopril, and enalapril, which have since become indispensable in clinical practice, underscoring the profound impact of their discovery. What makes this achievement even more remarkable is that it was accomplished in the absence of detailed chemical, kinetic, or structural insights into human ACE.

**Figure 4:**
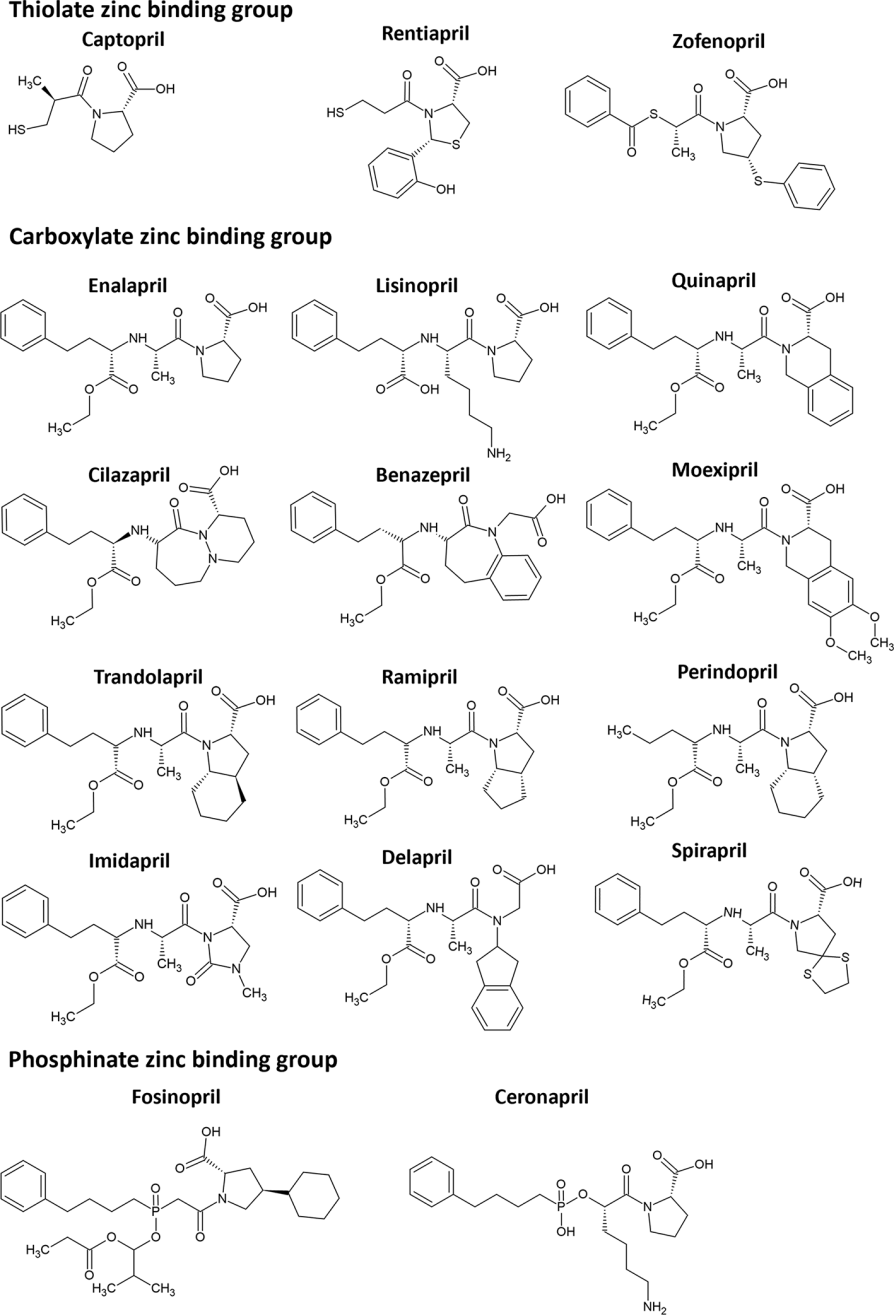
Current clinically used ACE inhibitors The chemical structures of 17 ACE inhibitors classified according to their zinc binding groups.

Currently there are 17 clinically used ACE inhibitors which can be stratified into three distinct chemical classes (Figure 4). The first class comprises thiolate compounds, such as captopril, which exert their effects through thiolate coordination to the zinc and additional hydrogen bonding to the carboxylate. While these compounds may elicit some undesired effects due to their broad reactivity, they also offer therapeutic benefits. The second class encompasses carboxylate compounds, including enalapril and lisinopril, known for their enhanced potency compared to captopril. The third class comprises phosphinate zinc binding group compounds like fosinopril, which have prolonged duration of action compared to the other two classes of ACE inhibitors.

The enduring legacy of ACE inhibitors underscores the transformative power of translational research and the critical interplay between basic science and clinical applications. Moving forward, further advancements in our understanding of ACE biology and drug design will enable the development of next-generation inhibitors with enhanced efficacy, safety, and specificity.

### Adverse effects of current ACE inhibitors

ACE inhibitors are a commonly prescribed class of medication known for their efficacy and relatively low incidence of adverse effects. However, approximately 20–25% of patients experience difficulties tolerating long-term treatment due to various undesired side effects, including, skin rash, increased blood potassium levels (hyperkalaemia), dizziness from excessive blood pressure reduction, headaches, and loss of taste. One of the most common adverse effects of ACE inhibitors is a persistent dry cough. In potentially life-threatening cases, which are often specific to a particular population group, ACE inhibitors can induce angioedema, which is characterized by swelling of the throat and tongue [24,25]. These adverse reactions are attributed to increased levels of BK or substance P, which stimulate vagal fibres [26]. Moreover, ACE inhibitors can lead to decreased aldosterone levels, contributing to hyperkalaemia in individuals with compromised kidney function.

Certain ACE inhibitors containing sulfhydryl groups, like captopril, have been associated with rash, neutropenia, and nephrotic syndrome, especially in patients with renal insufficiency [27,28]. Long-term ACE inhibitor use may also result in ACE inhibitor escape, where Ang II levels fail to normalize. This phenomenon could be facilitated by the hydrolysis of Ang I-12 by chymase, as observed in rodent studies where chronic ACE inhibitor treatment led to heightened chymase activity in the left ventricle mediated by the BK receptor [29].

Given the broad substrate specificity of ACE and the potential for other enzymes to metabolize Ang II, there is a growing need for next-generation ACE inhibitors that have been designed to target the catalytic site of cACE. These inhibitors aim to mitigate the adverse effects associated with current ACE inhibitors while enhancing their therapeutic efficacy. This approach will ultimately improve patient outcomes and enhance medication tolerability in the management of various cardiovascular conditions. Despite the aforementioned advances in the structural basis of ACE inhibition, the development of domain-selective ACE inhibitors has been limited, with none currently available for clinical use. The pursuit of such inhibitors remains promising as they could potentially mitigate unwanted effects and enable tailored treatments.

### New generation of domain specific ACE inhibitors

The application of molecular cloning techniques has revolutionized our understanding of ACE and its distinct domains. During the 1960s and 1970s, ACE was believed to comprise of a single polypeptide chain with a single active site, which guided the design of traditional ACE inhibitors. However, the discovery of the full-length ACE gene expression and the isolation of nACE and cACE have revealed valuable insights into the *in vivo* functions and synergies of these structurally similar yet functionally distinct domains [30]. Animal studies, particularly those utilizing transgenic mice expressing ACE with inactivated nACE or cACE, have shed light on the unique physiological roles of these domains [22]. It is now evident that nACE and cACE play distinct roles [21], albeit with minor differences in potency and pharmacokinetic properties.

Inhibitors specifically targeting cACE are anticipated to exert cardiovascular effects that are similar to those of current generation ACE inhibitors. However, cACE-specific inhibitors may have improved side effect profiles, which can be primarily attributed to reduced BK levels. Moreover, the potential therapeutic spectrum of cACE-selective inhibitors may differ from that of conventional ACE inhibitors, which typically inhibit both nACE and cACE. Despite the modest benefits observed with normal clinical doses of ACE inhibitors in slowing cardiovascular disease (CVD) end-organ damage, there is growing recognition of the incomplete blockade of RAAS as a contributing factor. A novel approach involves elevating plasma levels of Ac-SDKP, derived from thymosin β4, known for its anti-inflammatory and anti-fibrotic properties. Since Ac-SDKP degradation primarily relies on hydrolysis by nACE, selective inhibition of nACE could increase plasma Ac-SDKP levels, offering cardio- and reno-protective effects without excessive RAAS inhibition.

Preclinical evidence suggests that nACE-selective ACE inhibitors could enhance tolerance to bleomycin in cancer therapy and mitigate fibrosing lung diseases. Notably, highly selective nACE inhibitors may substantially elevate Ac-SDKP levels, providing protective effects without inducing hypotension, hyperkalaemia, or renal impairment associated with excessive RAAS inhibition.

**Figure 5:**
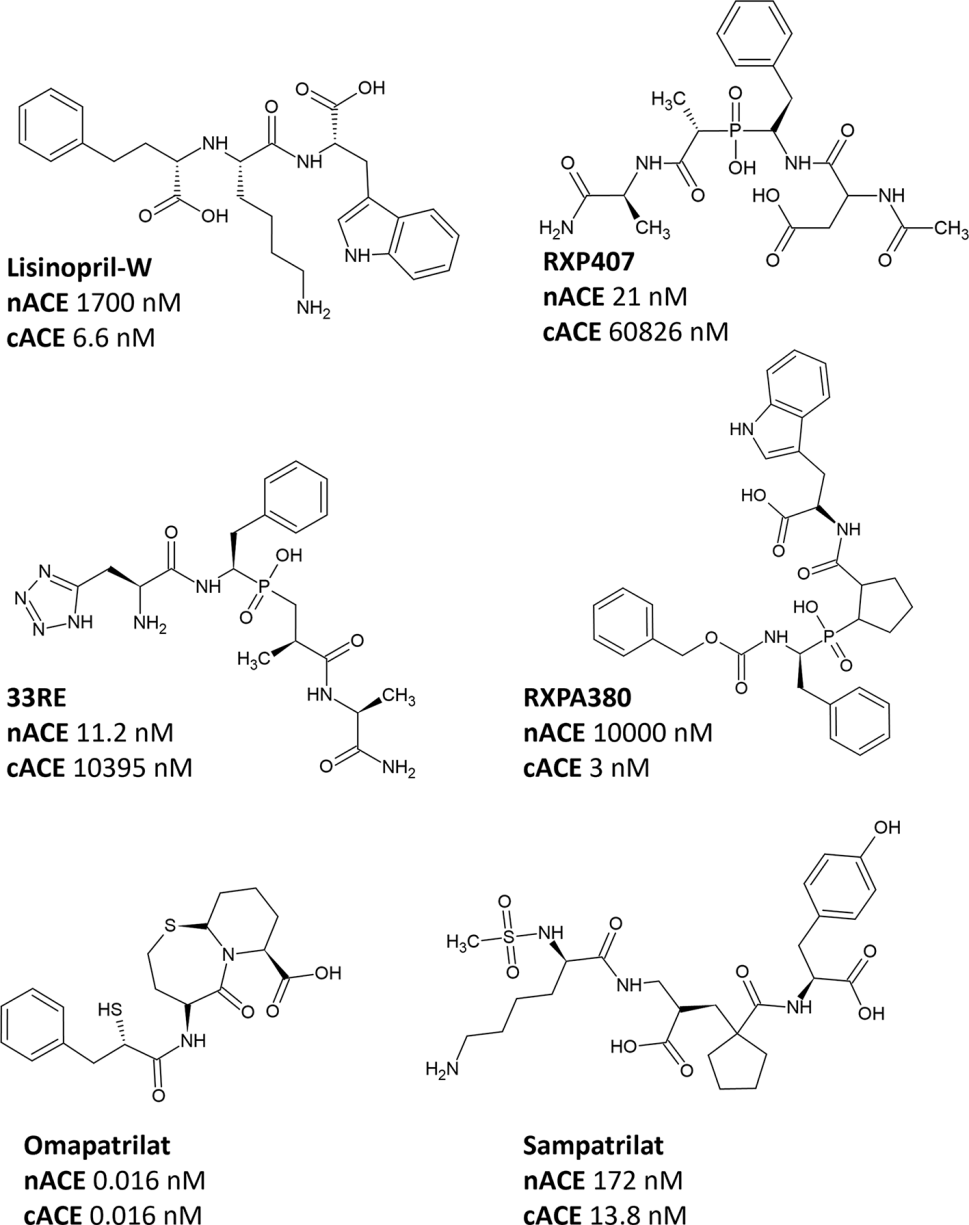
Structures of selective ACE inhibitors with *K*_i_ values for N-domain ACE (nACE) and C-domain ACE (cACE)

Currently, there are no commercially available domain-selective ACE inhibitors. However, experimental compounds with significant selectivity for one domain over the other have been identified [31–36] and the molecular basis of their inhibition with individual domains of ACE has been studied with the aid of high-resolution crystal structures using X-ray crystallography (Figure 5). The P1′ proline and P2′ tryptophan moieties of RXPA380 are largely responsible for its C-selectivity. The tryptophan makes favourable interactions with Val379 and Val380, which are replaced by a serine and threonine in nACE [37]. Insights from this work led to the development of lisinopril-tryptophan (Lis-W) where the P2′ proline of the drug lisinopril is replaced with a tryptophan [38]. Kinetic and crystal structure studies revealed that Lis-W was highly C-domain-selective and provided a molecular basis for the C-selectivity which was similar to that for RXPA380 [33]. Furthermore, Lis-W reduced blood pressure and angiotensin II levels similarly to conventional ACE inhibitors but without increasing BK levels in a hypertensive mouse model [39].

The phosphinic peptide RXP407 is highly selective for the nACE active site with a *K*_i_ value 2000-fold lower than that for cACE. The P2 aspartate and P2′ amidated alanine of RXP407 were shown to be largely responsible for its N-selectivity [31]. More recently, a fragment-based approach was used to produce analogues of RXP407 with different P2 functionalities. Replacing the P2 aspartate with an aminomethyl tetrazole yielded the compound 33RE, which was 1000-fold nACE selective [40]. Surprisingly, S2′ residues that do not interact directly with 33RE, contributed to the selective binding of the inhibitor based on mutagenesis of the S_2_′ residues in the nACE. This was confirmed using molecular dynamics; however, these residues were involved in key interactions between the subdomains keeping nACE in a closed ligand-bound conformation. This intersubsite cooperativity should be taken into consideration when designing domain-selective inhibitors for various therapeutic interventions.

Despite their potential, existing compounds like RXP407 and RXPA380 exhibit poor pharmacokinetic profiles, limiting their clinical utility [31]. These compounds (delivered as a single IV dose in rats) are rapidly cleared, unchanged, via renal excretion, probably because of their highly polar nature [20,41]. Compounds with similar pharmacological profiles, but substantially improved PK properties, are therefore required to deliver the benefits of a domain-selective ACE inhibitor. Thus, further research is warranted to develop compounds with improved pharmacokinetic properties while retaining domain selectivity. Compounds with enhanced pharmacological profiles are essential for realizing the therapeutic benefits of domain-selective ACE inhibitors.

### Dual ACE/NEP or vasopeptidase inhibitors

While ACE inhibitors and ARBs have been crucial in suppressing the RAAS, achieving optimal blood pressure reduction remains challenging. This has led to the development of vasopeptidase inhibitors, which target multiple structurally related peptidases involved in blood pressure and cardiovascular regulation [8].

A key system in this context is the natriuretic peptide (NP) system, which affects blood pressure, fluid and electrolyte balance, renal function, and cardiovascular health. Natriuretic peptides, including ANP, BNP, and CNP, induce natriuretic, diuretic, vasorelaxant, and antimitogenic effects to lower blood pressure and maintain fluid balance. The discovery and elucidation of the actions by which neprilysin (NEP) and its inhibitors exert these effects have revealed both the similarities and differences between the RAAS and the NP system [42–44]. Dual ACE and NEP inhibitor therapy has shown enhanced efficacy in animal models of heart failure and cardiomyopathy, leading to the development of orally active molecules that inhibit both ACE and NEP, known as dual inhibitors [45,46]. The structural similarities between ACE, NEP, and endothelin-converting enzyme (ECE-1), along with their overlapping substrate specificity, facilitated the design of molecules targeting two or three of these enzymes.

Early dual inhibitors were designed based on specific ACE and NEP inhibitors. Combining specific groups known to be important for NEP inhibition with structural elements from the initial ACE inhibitors led to the development of potent mercaptoacyl dipeptides with dual inhibitory activity [47,48]. Further refinement led to the development of omapatrilat, the first vasopeptidase inhibitor to enter clinical trials [49]. Omapatrilat aimed to block ACE-mediated Ang II formation and NEP-mediated degradation of vasodilatory natriuretic peptides [49–53].

However, large-scale clinical trials such as OVERTURE, OPERA, and OCTAVE failed to show omapatrilat’s superiority over traditional ACE inhibitors in reducing mortality or hospitalization for heart failure. Additionally, omapatrilat was associated with a higher risk of vasodilator-mediated adverse effects, particularly angioedema. These trials highlighted the increased incidence and severity of angioedema with omapatrilat compared with ACE inhibitors.

Despite extensive research, significant uncertainties remain regarding the physiology and pathophysiology of vasoactive peptide systems and their impact on cardiovascular function and diseases. It might not be necessary to leave both nACE and NEP free to degrade vasodilatory peptides. ACE primarily metabolizes BK, suggesting that nACE may sufficiently compensate for cACE in preventing harmful BK levels. Therefore, dual cACE-selective/NEP inhibitors may offer a promising alternative enhancing natriuretic peptide levels while blocking Ang II formation. A fixed-dose combination of Lis-W and the NEP inhibitor sacubitril reduced blood pressure and improved cardiac function in Ang II-dependent hypertensive mice, preserving BK metabolism and not increasing vascular permeability [54]. This combination could treat hypertension and heart failure without the adverse effects of angioedema.

**Figure 6:**
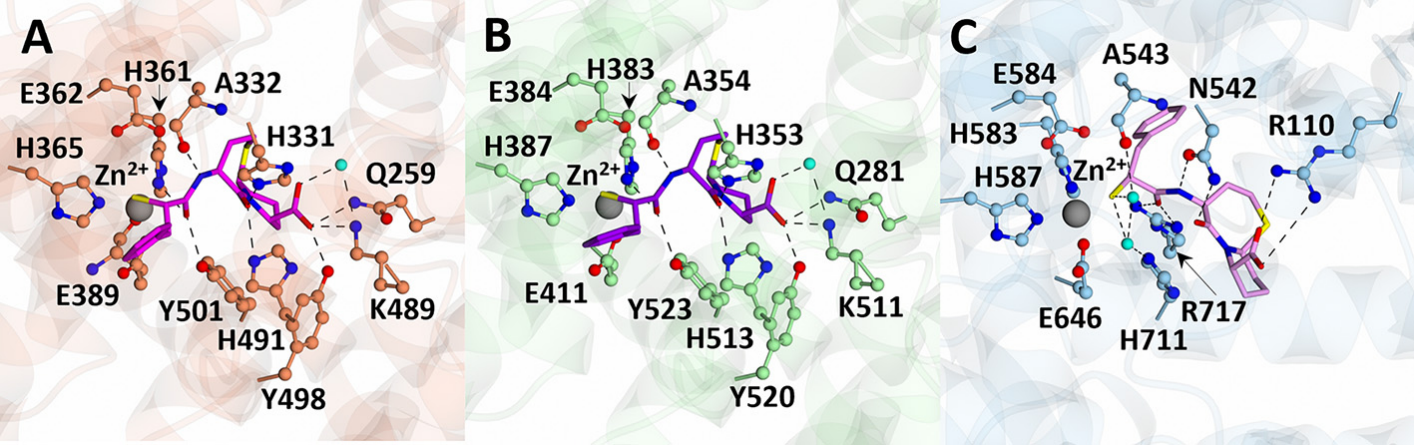
Structures of nACE (A), cACE (B), and NEP (C) in complex with the vasopeptidase inhibitor, omapatrilat The structures of nACE, cACE and NEP are shown in orange, light green, and light blue respectively. Omapatrilat in nACE, cACE, and NEP is shown in magenta, purple, and pink, respectively.

Recent insights from crystal structures of cACE and NEP in complex with dual inhibitors such as omapatrilat and sampatrilat [55–57] pave the way for the development of new leads with similar efficacy to omapatrilat but with improved side effect profiles (Figure 6). By targeting specific domains of ACE and NEP, these inhibitors may optimize cardiovascular therapy by enhancing the beneficial effects of natriuretic peptides while minimizing adverse effects associated with excessive RAAS inhibition.

### Domain co-operativity between individual domains of ACE

**Figure 7:**
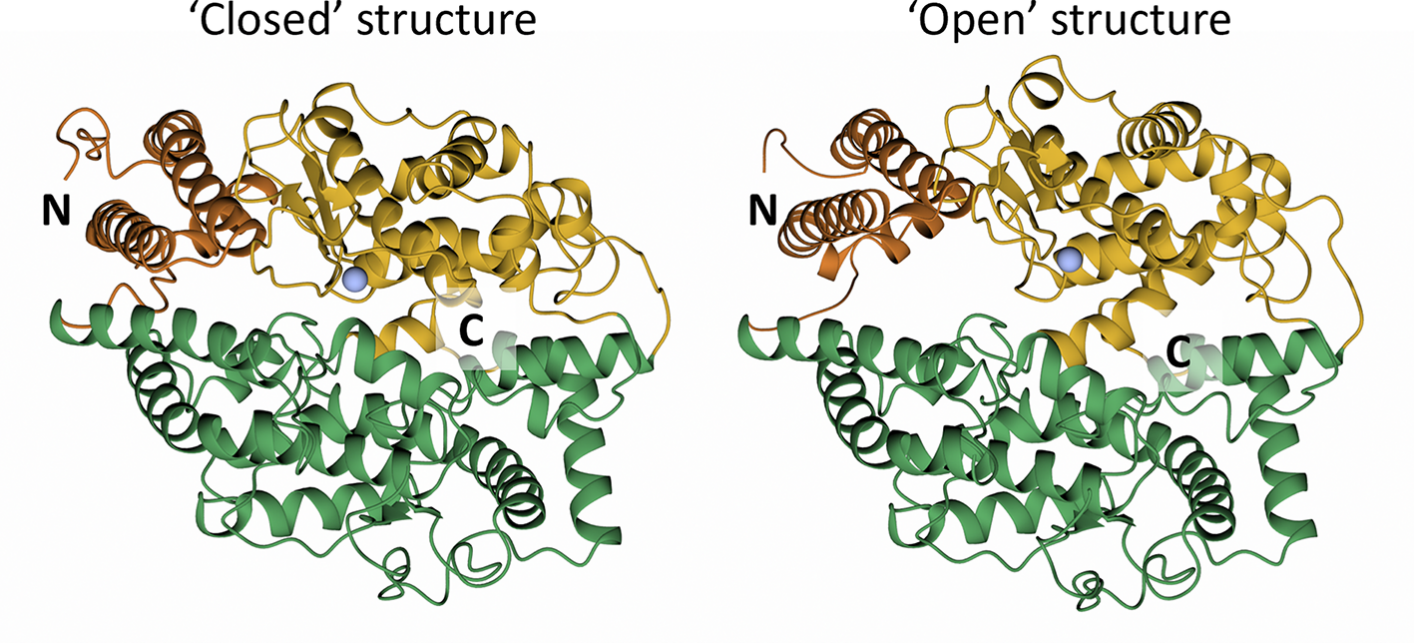
Crystal structures of ‘closed’ and ‘open’ nACE Sub-domain 1 is shown in yellow (The lid-like region in orange), and sub-domain 2 in green. The zinc ion is shown by the grey sphere.

Kinetic studies have revealed negative cooperativity between the N- and C-domains in the hydrolysis of substrates [12,58,59]. Other work suggests that proximity of the two domains affects substrate cleavage and shedding implying that the domains could be in an intimate orientation to allow for subtle allosteric or interdomain effects [60]. Structural studies have helped to elucidate the nature of these complex interactions and suggest that the transition from an open to closed conformation of each domain is initiated by substrate or inhibitor binding [40,61,62] (Figure 7). Site-directed mutagenesis of sites distal to the inhibitor suggest there is allosteric regulation that has a dramatic effect on inhibitor binding [40,61,63]. Research by Kost et al [64] and other groups showed that inhibitor binding to one domain can negatively affect the function of the second domain resulting in ACE homodimerization, phosphorylation on Ser1270, and activation of the c-Jun N-terminal kinase, thus leading to an increase in gene expression [64–67].

There may be some ‘cooperativity’ between the two domains of ACE, which could have significant effects on the pharmacological profile of domain-selective inhibitors [58,59,68,69]. In addition, mutations in nACE [70] cause increased ectodomain shedding in the C-terminal juxtamembrane region.

The majority of the structural information available for ACE is from crystal structures of the individual domains in the closed conformation bound to inhibitors. These structures do not shed light on the open conformation of the enzyme prior to inhibitor binding, or on the relative positioning of the N- and C-domains in somatic ACE. Thus, we only have a preliminary understanding of what determines the specificity of the individual domains of ACE, and the mechanisms through which the activities are regulated remains elusive. It is thus key to corroborate any domain-specific inhibitors with full-length somatic ACE. To achieve safe and effective treatments for diseases involving ACE and the RAS, it is important to further investigate the dimerization and cooperativity of ACE in order to understand the mechanisms of its intra- and inter-molecular interactions. This heralds an urgently needed shift from an active site-orientated perspective to a holistic view of the ACE ectodomain.

### Structural features of full-length ACE: cryo-electron microscopy studies

**Figure 8:**
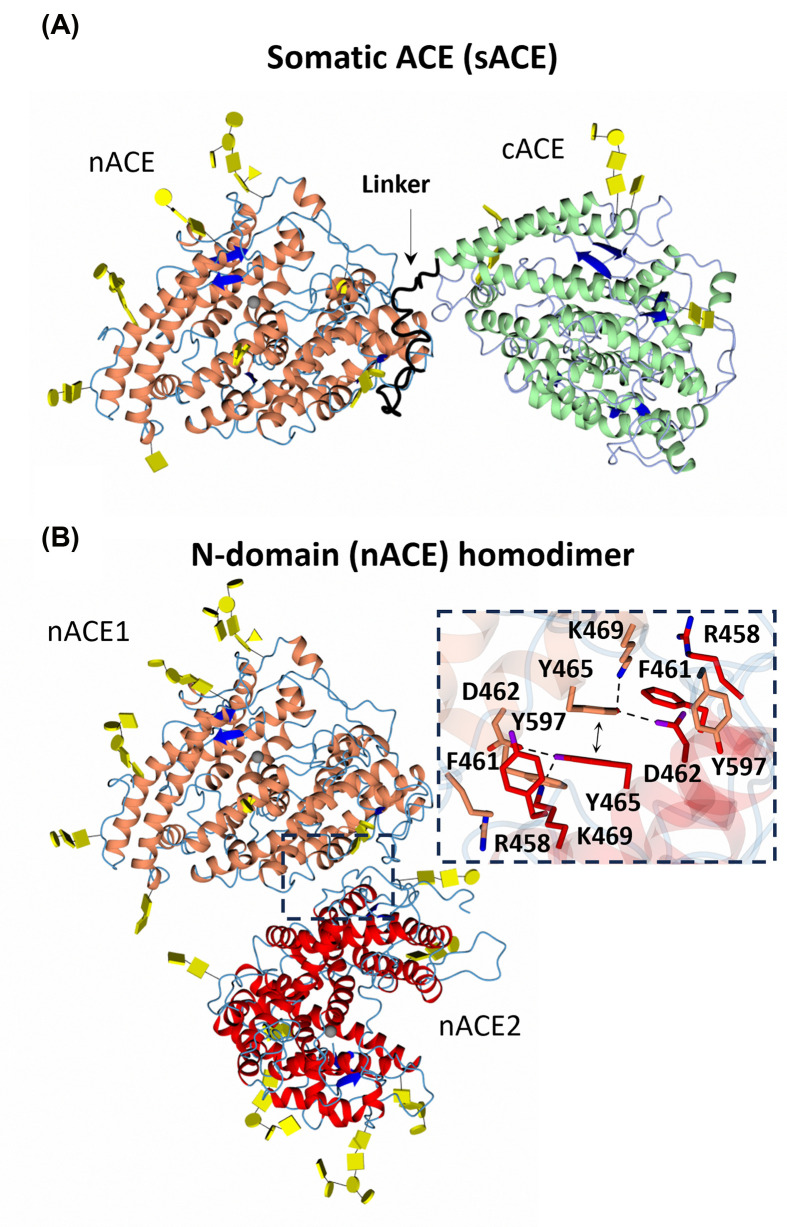
Cryo -EM structures of open sACE (A) and nACE homodimer (B) The α-helices of nACE and cACE are shown in orange and light green, respectively, and the linker region in black. The second molecule of the nACE homodimer is shown in red. β-strands are shown in blue. Glycosylated carbohydrates shown in yellow. The homodimer interface is shown in the dotted square. Dotted lines represent hydrogen bonding interactions, and the double arrow indicates π-stacking.

Recent advancements in cryo-electron microscopy (cryo-EM) offer a promising approach to overcome the limitations of X-ray crystallography in studying flexible and glycosylated proteins like ACE. Cryo-EM was effectively used by Lubbe et al. [71] to elucidate the structure of human ACE, focusing on its soluble form, which naturally occurs in bodily fluids. Unlike previous crystal structures that were truncated and lacked full glycosylation, the cryo-EM structures captured the entire ACE (the full-length enzyme with both domains intact) in its fully glycosylated and apo state. Their analysis revealed that ACE exists in both monomeric and dimeric forms, with the latter being a minor fraction. Despite challenges in particle identification and classification due to the protein’s small size and low signal-to-noise ratio, they successfully reconstructed high-resolution structures of both monomeric and dimeric ACE using cryo-EM [71] (Figure 8).

The cryo-EM structures revealed an open conformation for both nACE and cACE domains of ACE; in addition, the full-length structure continuously changed between the open and closed states providing insights into potential allostery and domain cooperativity. Importantly, they identified an allosteric site on nACE that could be targeted for the development of domain-selective ACE inhibitors. The study by Lubbe et al. [71] also shed light on the mechanism of sACE homodimerization, showing interactions between nACE surfaces and glycan-glycan interactions contributing to dimer formation. Dimerization was associated with increased flexibility in the interdomain linker and significant motion within and between the two domains, suggesting a role in modulating ACE activity and signalling.

The new cryo-EM structures of ACE provide valuable insights into its structure-function relationship. By elucidating its dynamics, domain cooperativity, and allosteric regulation, this study lays the groundwork for improved ACE inhibitor design and provides valuable insights into the physiological roles of ACE beyond its enzymatic activity.

### Design of allosteric inhibitors of ACE

Allosteric inhibitor design targeting exosites away from the zinc active site of ACE presents a promising approach for modulating ACE activity, with potential therapeutic implications. Traditional ACE inhibitors competitively bind to the zinc active site, effectively blocking its enzymatic activity. However, allosteric inhibition offers an alternative strategy by targeting distinct sites on the enzyme’s surface, away from the active site, thereby modulating its function without interfering directly with substrate binding. Such allosteric inhibitors offer several advantages over traditional competitive inhibitors, including potentially higher specificity, reduced risk of resistance development, and the ability to fine-tune enzyme activity without complete inhibition.

The allosteric control of peptidases and processes involved in zymogen activation was recently reviewed by Obaha and Novinec [72], and provides insight into the mechanisms that regulate peptidase activity and the opportunities for improved therapeutic intervention [72]. The allosteric activators of ACE2, minithixen and the antiprotozoan drug diminizene aceturate (DIZE), which target the hinge region of ACE2, caused a significant increase in enzyme efficiency. Moreover, DIZE revealed beneficial effects in different disease models, such as hypertension, atherosclerosis and diabetes [73].

One promising approach to allosteric inhibition of ACE involves targeting its exosites involved in protein–protein interactions with substrates or other regulatory molecules. There are two possible approaches. Firstly, the sites of ACE homo-dimerization on nACE (Figure 8B) could be targeted indirectly influencing ACE ectodomain shedding and gene expression [11,66]. Furthermore, ACE homodimerization activates an allosteric switch, and the nACE active site His361 is rotated away from the catalytic zinc. This conformational change together with that of Lys489 which is rotated out of the active site, suggest that dimerization inactivates the N-domain active site.

Secondly, exosites, located on the enzyme’s surface, often play crucial roles in substrate recognition, binding, and catalysis. By disrupting these interactions, allosteric inhibitors can modulate enzyme activity indirectly. The rich structural knowledge available for ACE and its inhibitors, complemented by the full-length cryo-EM structure, has allowed the identification of seven potential sites that could be targeted for allosteric modulation of ACE activity [71]. The use of CavityPlus predicted allosteric sites which can affect nACE, cACE, or both orthosteric sites. Site 1 allosterically affects the nACE active site, which can be explained by loop -2 and -3 which extend from the nACE surface to key active site residues (Lys489 and Tyr498).

Despite these advancements, several challenges remain in the design of allosteric inhibitors targeting ACE. The complex and dynamic nature of protein-protein interactions and conformational changes in ACE pose significant challenges for inhibitor design and optimization, including the use of soluble sACE instead of truncated domains for kinetic validation of allostery, and the need to ensure that only the intended catalytic site is inhibited and not both domains.

Additionally, achieving selectivity and potency in allosteric inhibitors remains a formidable task, as these compounds must compete with other regulatory molecules for binding to the enzyme surface. Rational design approaches, combined with high-throughput screening, computational modelling, and *in vitro* validation are essential for identifying allosteric inhibitors with the desired pharmacological properties.

Moreover, the development of allosteric inhibitors targeting ACE requires thorough characterization of their pharmacokinetic and pharmacodynamic properties to ensure efficacy and safety *in vivo*. Preclinical studies in animal models of hypertension and cardiovascular disease are essential for evaluating the therapeutic potential of allosteric inhibitors and optimizing their pharmacological properties.

## Conclusions

The development of next-generation ACE inhibitors represents a pivotal advancement in cardiovascular therapy and the treatment of fibrotic disorders. Current ACE inhibitors typically inhibit both cACE and nACE, leading to a persistent cough and angioedema. Thus, the design of selective ACE inhibitors targeting either cACE or nACE remains a clinically important goal.

Selective inhibition of cACE holds promise for reducing adverse effects related to elevated levels of BK and substance P. Conversely, nACE-specific inhibitors are prospective therapeutics for reducing inflammation and fibrosis in cardiac, kidney, and lung tissue without influencing blood pressure. New strategies in drug design have introduced the concept of multitarget compounds, aiming to address multiple aspects of disease pathology simultaneously.

The availability of high-resolution crystal structures of cACE and nACE facilitates rational structure-based drug design. While several crystal structures with compounds inhibiting either the human cACE and nACE have been solved, only a few structures of different inhibitors in complex with both nACE and cACE have been published. Analysis of these complexes revealed that inhibitor selectivity for nACE and cACE is driven by discrete differences in distant amino acids of the catalytic centre in the S2 and S2′ subsites.

Crystal structure complexes of moderately cACE-selective inhibitors showed that molecules occupy an unusual binding pose and possess interactions with the non-prime S3 binding site. Furthermore, multiple ligand binding modes were observed in certain complexes, suggesting the potential for developing domain-selective and allosteric inhibitors targeting non-conserved regions of the ACE-binding cavity.

While multiple ligand binding in one enzymatic centre may be unlikely under physiological conditions, understanding these non-prime binding modes could guide the development of novel inhibitors. Additionally, inhibitors bound in regions distant from the catalytic centre could serve as a guide to identify target areas inside the binding cavity. In contrast, complexes of nACE with inhibitors did not indicate multiple molecule binding modes, possibly due to challenges in interpreting electron density maps. However, further exploration of these complexes may reveal insights into developing selective nACE inhibitors.

Overall, the elucidation of crystal structures and understanding of inhibitor binding modes offer valuable insights for the rational design of next-generation ACE inhibitors. By targeting specific domains or exosites distal to the active site, these inhibitors hold the potential to minimize adverse effects while maximizing therapeutic efficacy. Continued research in this field promises to advance cardiovascular therapy and the treatment of fibrotic disorders, improving patient outcomes and quality of life.

## Open Access

Open access for this article was enabled by the participation of University of Bath in an all-inclusive *Read & Publish* agreement with Portland Press and the Biochemical Society under a transformative agreement with JISC.

## Abbreviations

ACE: angiotensin‐1 converting enzyme
Ang I: angiotensin I
Ang II: angiotensin II
cACE: angiotensin‐1 converting enzyme C-domain
nACE: angiotensin‐1 converting enzyme N-domain
RAAS: renin–angiotensin–aldosterone system
sACE: somatic angiotensin‐1 converting enzyme
tACE: testis angiotensin‐1 converting enzyme
